# Impact of Nuclear Interleukin-1 Alpha and EGFR Expression on Recurrence and Survival Outcomes in Oral Squamous Cell Carcinomas

**DOI:** 10.1155/2019/5859680

**Published:** 2019-06-19

**Authors:** Anand Rajan, Katherine N. Gibson-Corley, Allen B. Choi, Georgina K. Ofori-Amanfo, Patrick Ten Eyck, Madelyn Espinosa-Cotton, Steven M. Sperry, Andrean L. Simons

**Affiliations:** ^1^Department of Pathology, University of Iowa Hospitals and Clinics, Iowa City, IA 52242, USA; ^2^Institute for Clinical and Translational Science, University of Iowa Hospitals and Clinics, Iowa City, IA 52242, USA; ^3^Free Radical and Radiation Biology Program, Department of Radiation Oncology, University of Iowa Hospitals and Clinics, Iowa City, IA 52242, USA; ^4^Department of Otolaryngology–Head and Neck Surgery, University of Iowa Hospitals and Clinics, Iowa City, IA 52242, USA; ^5^Holden Comprehensive Cancer Center, University of Iowa Hospitals and Clinics, Iowa City, IA 52242, USA

## Abstract

Interleukin-1 alpha (IL-1*α*) is a pleiotropic cytokine involved in inflammation and immune response and is upregulated in many solid tumors including head and neck squamous cell carcinomas. Although IL-1*α* expression is generally associated with poor prognosis, the implications of the subcellular localization of IL-1*α* expression in patient outcomes are poorly understood. This study is aimed at investigating the prognostic value of nuclear and cytoplasmic immunohistochemical IL-1*α* expression in oral squamous cell carcinomas (OSCCs). Tissue microarrays containing 146 OSCCs were analyzed for IL-1*α* and epidermal growth factor receptor (EGFR) expression by immunohistochemistry. IL-1*α* and EGFR expression scores were correlated with clinicopathological parameters and survival outcomes. IL-1*α* expression was observed in the nuclear and/or cytoplasmic compartments in 98% of evaluable tumors and 78% of tumors expressed IL-1*α* in both compartments. There were no differences observed in overall survival or progression-free survival between high, moderate, or negative IL-1*α* nuclear/cytoplasmic expression scores. When IL-1*α* nuclear/cytoplasmic expression scores were stratified by positive or negative EGFR expression, tumors with a combined EGFR-positive and high nuclear IL-1*α* expression profile were significantly more likely to possess perineural invasion and were significantly associated with a high risk of tumor recurrence and worse progression-free survival compared to all other EGFR and combined IL-1*α*/EGFR expression profiles. Altogether, nuclear IL-1*α* expression may enhance the prognostic value of EGFR in OSCC and warrants further study as a prognostic biomarker for recurrence.

## 1. Introduction

The IL-1 pathway plays a plays a critical role in the regulation of immune and inflammatory responses to infections and sterile insults [1]. IL-1 signaling is also frequently upregulated in many solid tumor types including head and neck squamous cell carcinomas (HNSCCs) [2]. Although increased IL-1 signaling is typically associated with poor prognosis in cancer patients [3–5], the role of IL-1 signaling in cancer is controversial since IL-1 plays roles in both tumor promotion via the expression of genes involved in tumor survival, angiogenesis, metastasis, and immune cell recruitment [6–8] and tumor suppression via increased natural killer (NK) cell activity and enhanced Th1-mediated immunity [9, 10].

The IL-1 pathway is triggered when the ligands IL-1*α* and IL-1*β* bind to IL-1 receptor type I (IL-1R1). Upon ligand binding, the receptor forms a heterodimer with IL-1 receptor accessory protein (IL-1RAcP), which leads to the recruitment of the cytosolic coadaptor protein myeloid differentiation primary response gene 88 (MyD88) via its toll-like-interleukin-1 receptor (TIR) domain, followed by recruitment of IL-1 receptor-associated kinases and TNF Receptor Associated Factor 6 (TRAF6) [11]. These signaling events activate nuclear factor kappa-light-chain-enhancer of activated B cells (NFkB) and mitogen-activated protein kinases (MAPK) signaling leading to the expression of IL-1 target genes including activating IL-1 ligands (IL-1*α* and IL-1*β*) which activate and reinforce a positive feed-forward loop and sustained release of cytokines [11].

Of the activating ligands in the IL-1 family, IL-1*β* is the most studied. IL-1*β* is initially translated into an immature pro-IL-1*β* and cleaved into its active form by caspase-1 and released from the cell [11]. IL-1*α* is less studied and has a different biological role than IL-1*β*. IL-1*α* also exists as a precursor (pro-IL-1*α*) and can be cleaved by the calcium-activated neutral protease calpain into a 17-kDa C-terminal component, known as “mature” IL-1*α*, and a 16-kDa N-terminal propiece (ppIL-1*α*) [12]. Both pro-IL-1*α* and the mature IL-1*α* are active and are able to bind to IL-1R1 and trigger signaling [12]. Pro-IL-1*α* also contains a nuclear localization signal (NLS) not present in the mature form, which is retained in the ppIL-1*α* after cleavage of pro-IL-1*α* [13]. This allows both pro-IL-1*α* and ppIL-*α* to translocate to the nucleus. Nuclear localization of IL-1*α* (i.e., pro-IL-1*α* and ppIL-1*α*) is believed to be functionally important, due to the ability of these entities to bind DNA [14] and activate NFkB and specificity protein 1 (Sp1) which play important roles in cell proliferation [15].

Previous work has shown that HNSCC tumors have increased mRNA expression levels of IL-1*α* and IL-1*β* compared to adjacent normal tissue [6] and that tumors with high IL-1*α* gene expression and protein secretion are associated with the development of distant metastasis in HNSCC patients [5]. Despite these previous observations, little is known about the* in situ* tumor expression of IL-1*α* in HNSCCs, the subcellular distribution of IL-1*α* in HNSCC tumors, and the prognostic significance of IL-1*α* localization in HNSCC patients. Additionally, EGFR is well known as a prognostic marker in HNSCC [16] and a crosstalk relationship has been previously reported between the EGFR and IL-1 pathways [17, 18]. The goals of this study are to (1) characterize the subcellular distribution of IL-1*α* in OSCCs and (2) determine the prognostic significance of IL-1*α* subcellular localization and combined EGFR/IL-1*α* expression in OSCC patients.

## 2. Materials and Methods

### 2.1. Tissue Microarrays (TMAs)

Formalin-fixed paraffin-embedded tumor samples of patients were obtained from the archives of the Department of Pathology at the University of Iowa Hospitals and Clinics. TMAs were constructed using 3-6 morphologically representative tumor regions (1 mm) chosen from 146 carcinomas from the oral cavity. The 146 cases were surgical resection specimens and chosen selectively to ensure a mixture of patients with and without recurrence, with and without node metastases or positive margins, young and old ages, and smokers and nonsmokers from a population of 421 patients spanning 10 years of time (2005-2014). After generating a list of 266 patients proposed for inclusion in the TMA, only 146 were included in the TMA block construction due to paraffin-embedded formalin-fixed tissue block availability and quality and resource constraints. Chi-square analysis of the T stage distribution of the patients in the larger database (T1/T2/T3&4: 31/29/39%) showed no statistically significant difference from the patient cohort included in the study (29/29/40%). None of the patients included in the TMA underwent prior radiation or chemotherapy. Four *μ*m sections were obtained from the TMAs on poly-L-lysine-coated glass slides. Routine hematoxylin and eosin (H&E) sections were reviewed to confirm the original diagnosis.

### 2.2. Immunohistochemistry (IHC)

Antigen retrieval was performed on freshly cut sections in a decloaking chamber for 5 min at 125°C in TRIS buffer (pH 9.0). Endogenous peroxidase was blocked by incubation with 3% peroxide at room temperature for 2 min. For IL-1*α* staining, human specific IL-1*α* antibody (ab9614, Abcam) was applied at 1:250 in Dako diluent for 2 h at room temperature. The peptide sequence used to raise this antibody is SAPFSFLSNVKYNFMRIIKYEFILNDALNQSIIRANDQYLTAAALHNLDEAVKFDMGAYKSSKDDAKITVILRISKTQLYVTAQDEDQPVLLKEMPEIPKTITGSETNLLFFWETHGTKNYFTSVAHPNLFIATKQDYWVCLAGGPPSITDFQILENQA (amino acids 113-271) and therefore recognizes both full length/pro-IL-1*α* (amino acids 1-271) and mature IL-1*α* (amino acids 113-271). EGFR immunostaining was performed with antibody (H11, Dako) at 1:200 dilution. Bound antibody was detected using Envison™ + HRP, rabbit (Dako North America) for 30 min at room temperature followed by incubation with diaminobenzidine substrate (DAB) for 5 min at room temperature. HPV status was determined by p16 expression [19, 20]. After completion of IHC, slides were stored at room temperature and a virtual scanned copy of the TMA slides was kept for further reference.

### 2.3. Quantification of IL-1*α* and EGFR Staining

IL-1*α* immunoreactivity was evaluated by KGC using an Olympus BX53 microscope with an Olympus DP72 camera. Human kidney and spleen were used as positive and negative controls for IL-1*α* expression, respectively. Given that IL-1*α* expression varied in both the nuclei and cytoplasm, IL-1*α* expression was scored separately for the nuclei (N) and cytoplasm (C) on a scale from 0 to 2, with 0 representing no staining, 1 low/weak staining, and 2 strong/intense staining. The percentage of tumor cells with positive staining was determined by scoring 10 microscopic fields of 100 tumor cells each. Quantitative evaluation of EGFR staining was performed by AR according to Gamboa-Domingez et al. and Modern Pathology, 2004 [21], using a semiquantitative score (0-3+) where 0 represents no staining or membranous positivity in <10% neoplastic cells (negative) and 1, 2, and 3 represent weak, moderate, and strong membranous immunopositivity in >10% neoplastic cells.

### 2.4. Statistical Analysis

The associations between IL-1*α* and/or EGFR expression with clinicopathological features such as sex, age, smoking history, tumor site, pathological TNM classification (UICC 7th), differentiation (well/moderately/poor), perineural, lymphovascular, and bone invasion were tested using the generalized linear modeling (GLM) framework and the Kruskal-Wallis test. Differences in survival outcomes (overall survival [OS] and progression-free survival [PFS]) were plotted using the Kaplan-Meier method while estimates for the group hazard ratios were obtained using Cox proportional hazards (PH) modeling. OS is defined as the length of time from the date of diagnosis that the patients remain alive. PFS is defined as the time from diagnosis to disease progression or death from any cause. All testing was performed on the univariate level and unadjusted for multiple comparisons. Differences between survival curves were compared using the log-rank test. A* p*-value below 0.05 was considered statistically significant. All analyses were completed using SAS 9.4.

## 3. Results

### 3.1. Patient Characteristics

A total of 146 patient samples were included in the OSCC TMA, of which 141 showed interpretable IL-1*α* and EGFR immunostaining. Reasons for lost samples included loss/absence of tissue in the TMA section during IHC processing. The baseline characteristics for these patients are summarized in Table 1. Of the 141 patients, 82 patients were males with an average age of diagnosis of 58 years, and 59 patients were female with an average age of diagnosis of 66 years (Table 1). Smoking histories were reported in most patients with 56 (40%) active smokers, 13 (9%) that had quit smoking for less than 10 years, 17 (12%) that quit smoking for more than 10 years, and 6 (4.3%) tobacco chewers (Table 1). Males made up the majority (78%) of the active smokers and females made up the majority of the patients with no smoking history (68%, (data not shown)). Majority of tumor sites represented in the TMA were from the oral tongue, N0 (52%), and moderately differentiated (64%) (Table 1). Approximately 78 (55%) patients received adjuvant radiotherapy and 22 (16%) patients received chemotherapy following surgery (Table 1). Only 5 of the evaluable tumors were HPV-positive as detected by p16 expression (data not shown).

### 3.2. IL-1*α* Expression

**Figure 1:**
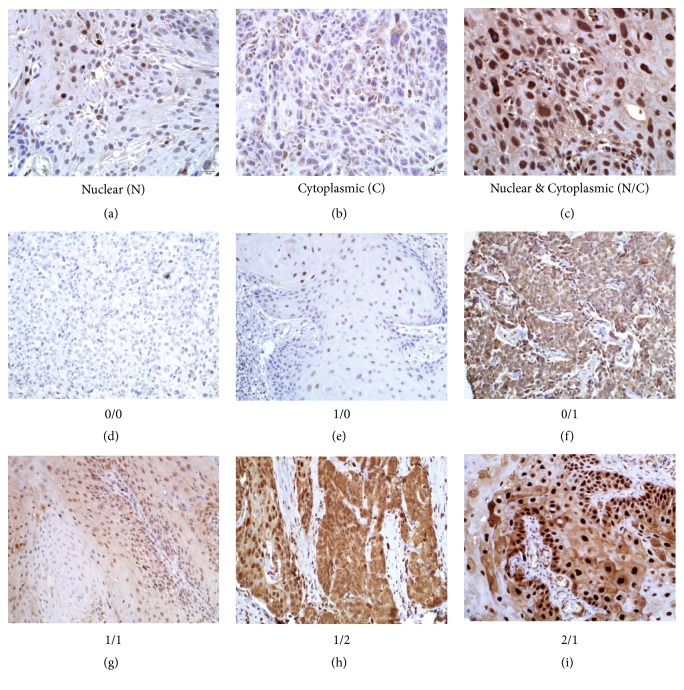
Representative examples of nuclear and cytoplasmic IL-1*α* immunostaining in OSCC and expression scores. (a) IL-1*α* expression in the nuclei; (b) IL-1*α* expression in the cytoplasm; and (c) IL-1*α* in both the nuclei and cytoplasm. (d) negative nuclear and cytoplasmic IL-1*α* expression; (e) moderate nuclear and negative cytoplasmic IL-1*α* expression; (f) negative nuclear and moderate cytoplasmic IL-1*α* expression; (g) moderate nuclear and cytoplasmic IL-1*α* expression; (h) moderate nuclear and strong cytoplasmic IL-1*α* expression; (i) strong nuclear and moderate cytoplasmic IL-1*α* expression. N: nuclear; C: cytoplasmic.

IL-1*α* expression was detected in the cytoplasm and/or nucleus in at least 1 of 3-6 cores for the vast majority of patients (98%). Examples of IHC images of primarily nuclear, primarily cytoplasmic and combined nuclear/cytoplasmic IL-1*α* immunoreactivity are shown in Figures 1(a)–1(c), respectively. Combined nuclear/cytoplasmic IL-1*α* scores were generated for each patient based on the intensity (negative [0], moderate [1], and strong [2]) of IL-1*α* staining in each compartment. Tumors were assigned IL-1*α* nuclear/cytoplasmic (N/C) expression profile scores including 0/0: negative nuclear and cytoplasmic IL-1*α* expression (Figure 1(d)); 1/0: moderate nuclear and negative cytoplasmic IL-1*α* expression (Figure 1(e)); 0/1: negative nuclear and moderate cytoplasmic IL-1*α* expression (Figure 1(f)); 1/1: moderate nuclear and cytoplasmic IL-1*α* expression (Figure 1(g)); 1/2: moderate nuclear and strong cytoplasmic IL-1*α* expression (Figure 1(h)); and 2/1: strong nuclear and moderate cytoplasmic IL-1*α* expression (Figure 1(i)). An IL-1*α* expression profile of 1/1 represented the majority (n=81, [58%]) of tumors in the TMA (Table 2).

### 3.3. Correlation of IL-1*α* Expression with Clinicopathologic Parameters

**Figure 2:**
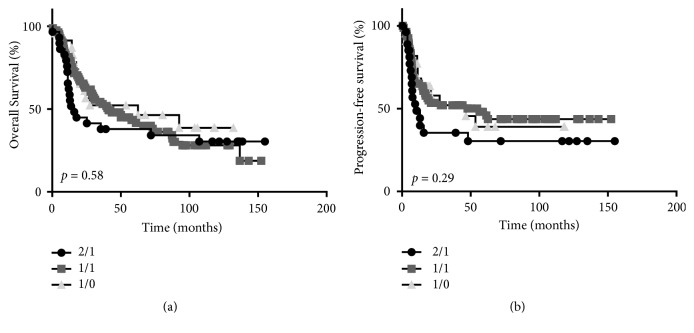
Prognostic impact by IL-1*α* expression score. Shown are Kaplan-Meier estimates of overall survival (a) and disease-free survival (b) according to IL-1*α* expression score.

There were no differences in nuclear or cytoplasmic IL-1*α* expression scores based on sex or age (Table 1). However significant differences in both nuclear and cytoplasmic IL-1*α* expression cores were observed based on smoking history, tumor site, T stage, N stage (cytoplasmic only), differentiation, perineural and lymphovascular invasion (cytoplasmic only), bone invasion, and the number of patients that received radiotherapy or chemotherapy (Table 1). There were not enough HPV-positive tumors to assess differences in IL-1*α* expression. Since the IL-1*α* expression profiles 1/1, 2/1, and 1/0 represented the majority of tumors in the TMA (Table 2), we further analyzed the association of these expression profiles with clinicopathological features of the represented OSCC patients. We found no differences in overall survival (*p*=0.27) or progression-free survival (*p*=0.29) with respect to these 3 major IL-1*α* expression profiles (Figures 2(a) and 2(b)).

### 3.4. EGFR Expression

**Figure 3:**
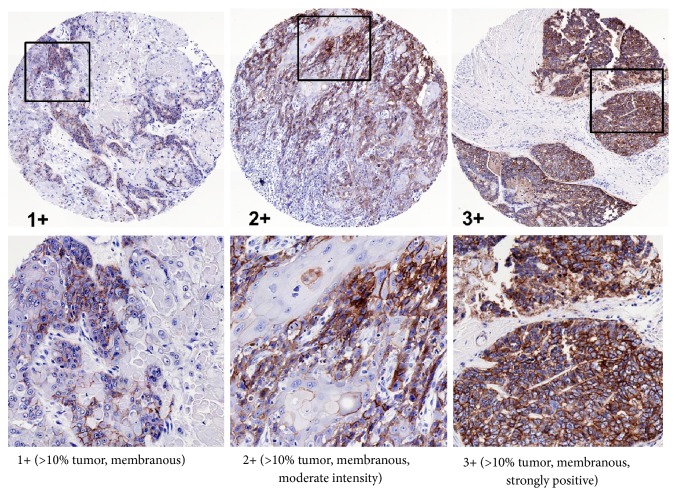
Representative examples of EGFR immunostaining and expression scores in OSCCs.

**Figure 4:**
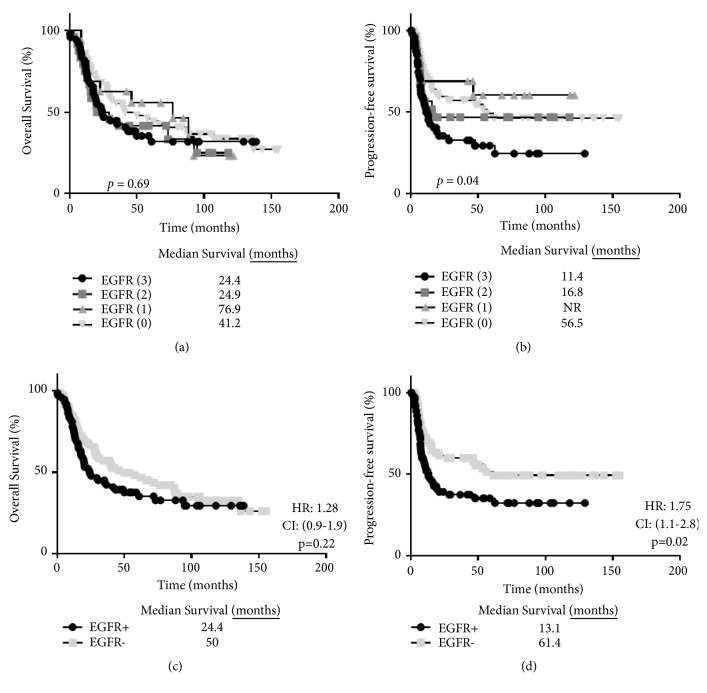
Prognostic impact by EGFR expression score. Shown are Kaplan-Meier estimates of overall survival (a, c) and disease-free survival (b, d) according to EGFR expression score. EGFR+ and EGFR- in (c) and (d) represent combined EGFR (3) + EGFR (2) and EGFR (1) + EGFR (0) scores, respectively. NR: not reached.

We next examined the role of EGFR expression in survival outcomes in OSCC patients. Examples of EGFR expression scores are shown in Figure 3. EGFR expression was observed in 61% of the tumors with strong (score of 3) expression in 34%, moderate (score of 2) expression in 16%, low (score of 1) expression in 11%, and no (score of 0) expression in of 39% of tumors (Table 3). There were no differences observed in overall survival (*p*=0.69) according to EGFR scores (Figure 4(a)); however significant differences were observed in progression-free survival (*p*=0.04) with higher EGFR expression being associated with worse progression-free survival (Figure 4(b)). A comparison of combined strong (3)+moderate (2) EGFR (designated as EGFR+) expression and combined low (1)+no (0) EGFR (designated as EGFR-) expression also showed a significant difference in progression-free survival (*p*=0.02) (Figure 4(d)) but not overall survival (*p*=0.22) (Figure 4(c)). These results support prior reports that EGFR expression is a strong predictor of progression-free survival in HNSCC patients [16, 22].

### 3.5. Correlation of Combined EGFR and IL-1*α* with Patient Outcomes

**Figure 5:**
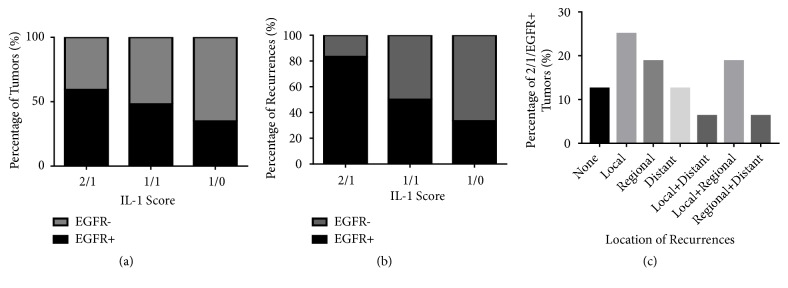
Recurrence rates by combined EGFR/IL-1*α* scores. Shown are the percentage of tumors (a) and percentage of tumor recurrences (b) based on combined EGFR and IL-1*α* expression scores. Location of recurrences in 2/1/EGFR+ tumors are shown in (c).

**Figure 6:**
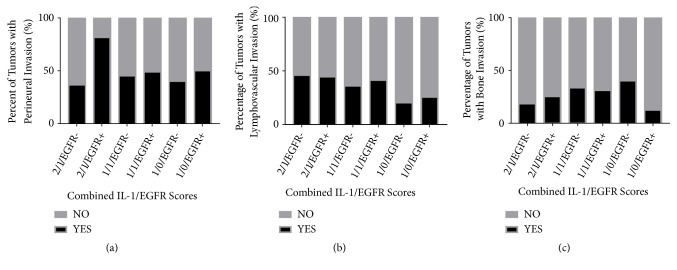
Invasion rates by combined EGFR/IL-1*α* scores. Shown are the percentages of tumors with perineural invasion (a), lymphovascular invasion (b), and bone invasion (c) by combined EGFR and IL-1*α* expression scores.

We next evaluated if differences in nuclear/cytoplasmic IL-1*α* expression scores altered the predictive value of EGFR. There were no significant differences (*p*=0.31) in EGFR expression in tumors with 1/0, 1/1, and 2/1 IL-1*α* expression profiles (Figure 5(a)). Tumors with a high nuclear/moderate cytoplasmic IL-1*α* expression profile (2/1) combined with a moderate/strong (2/3) EGFR expression score, which was designated as 2/1/EGFR+, were significantly (*p*=0.0058) more likely to experience tumor recurrence compared to the other IL-1*α*/EGFR expression profiles (Figure 5(b)). We also found a significant interaction (p=0.02) between the 2/1 IL-1*α* profile score and EGFR+ expression (Figure 5(b)) suggesting that this particular 2/1/EGFR+ expression profile could be considered a predictor of tumor recurrence. The high rates of recurrences observed in 2/1/EGFR+ tumors encompassed local, regional, and distant sites alone and in combination (Figure 5(c)). Additionally, 2/1/EGFR+ tumors displayed significantly higher rates of perineural invasion (p<0.0001) compared to the other IL-1*α*/EGFR expression profiles (Figure 6(a)). There were no differences among the IL-1*α*/EGFR expression profiles with respect to lymphovascular invasion (Figure 6(b)) and bone invasion (Figure 6(c)).

**Figure 7:**
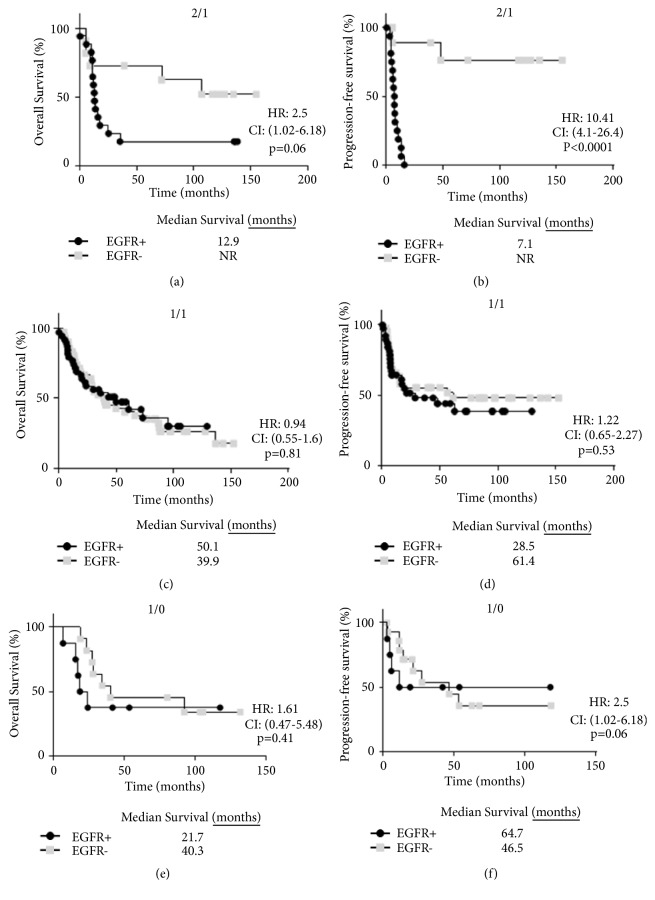
Prognostic impact by combined EGFR/IL-1*α* expression scores. Shown are Kaplan-Meier estimates of overall survival (a,c,e) and progression-free survival (b,d,f) according to EGFR expression scores and 2/1 (a,b), 1/1 (c,d) and 1/0 (e,f) IL-1*α* scores. HR: hazard ratio, CI: 95% confidence interval. NR: not reached.

Lastly, we found that patients with 2/1/EGFR+ tumors trended toward worse overall survival compared to 2/1/EGFR- tumors although this association did not reach statistically significance (*p*=0.06) (Figure 7(a)). However, 2/1/EGFR+ tumors were significantly associated with worse progression-free survival compared to 2/1/EGFR- tumors (*p*<0.0001) (Figure 7(b)). EGFR expression did not affect survival outcomes in 1/1 (Figures 7(c) and 7(d)) or 1/0 tumors (Figures 7(e) and 7(f)). Compared to patients with an overall EGFR- expression profile (median survival = 61.4 months, Figure 4(d)), patients specifically with 2/1/EGFR- tumors appeared to have the most favorable progression-free survival outcome (median survival=not reached) out of all of the other profiles (Figure 7(b)). Furthermore, we confirmed in Table 4 that adjuvant chemotherapy and/or radiation treatment did not influence the differences in progression-free survival observed with 2/1/EGFR+ versus 2/1/EGFR- tumors (Figure 7(b)). Altogether, these data suggest that high nuclear IL-1*α* expression in combination with moderate/high EGFR expression may be associated with worse outcomes in OSCC patients.

## 4. Discussion

The results from this study imply that nuclear IL-1*α* expression may enhance the prognostic value of EGFR with respect to progression-free survival. EGFR is already well known as a prognostic indicator in HNSCCs; therefore the question remains of what role does nuclear IL-1*α* play in EGFR signaling? IL-1*α* has been reported to be associated with poor prognosis in a wide range of cancers [7, 23–26]. In HNSCCs, gene expression, tumor cell secretion, and circulating levels of IL-1*α* have all been associated with tumor progression and distant metastasis [5–7]. On the other hand IL-1*α* is involved in antitumor immunity via increased natural killer (NK) cell activity, dendritic cell activity, and enhanced Th1-mediated immunity [9, 27–29]. These 2 opposing properties create a controversy surrounding the potential long-term clinical effectiveness of IL-1 inhibitors for cancer therapy. The additional presence of nuclear IL-1*α* further complicates this field since the activity of nuclear IL-1*α* is independent of the IL-1R1. For these reasons, little/no attention has been placed on the* in situ* analysis of IL-1*α* protein expression and the prognostic implications of IL-1*α* subcellular location.

In this study we have found that IL-1*α* is expressed in the vast majority of OSCC tumors and that IL-1*α* is expressed in both the nuclear and cytoplasmic components (Figure 1). We observed predominant nuclear expression of IL-1*α* since most of the IL-1*α* expressing tumors had either nuclear only or nuclear+cytoplasmic expression (Table 2). Only 4 tumors had cytoplasmic only staining highlighting the importance of nuclear IL-1*α* (i.e., pro-IL-1*α* and/or IL-1*α* propiece (ppIL-1*α*)) in tumor cells. In support of this observation, prior studies have also shown that pro-IL-1*α* and ppIL-1*α* appear to be predominantly intranuclear in IL-1*α*-expressing or IL-1*α*-transfected cells [30–33]. Based on the particular IL-1*α* antibody (ab9614, Abcam) used in these studies, the nuclear IL-1*α* detected is likely pro-IL-1*α* and not ppIL-1*α*. To date it is not clear what role nuclear IL-1*α* plays since some studies have reported that nuclear IL-1*α* inhibits cell proliferation [30, 31] and triggers apoptosis [32]; and in other studies nuclear IL-1*α* promotes cell proliferation [34]. It is possible that differences in experimental techniques and cell models may explain these contradicting reports. Nevertheless, we find no prognostic value in IL-1*α* subcellular localization (Figures 2(a) and 2(b)) in this cohort of OSCC patients. However, when we take into consideration EGFR expression we found unexpected but interesting results. When separating patients from the 2/1, 1/1, and 1/0 IL-1*α*-expressing groups into EGFR+ and EGFR- subgroups, we uncover a subset of tumors with a 2/1/EGFR+ profile that demonstrates significantly increased rates of perineural invasion compared to all other IL-1*α*/EGFR expression profiles (Figure 6(a)). The presence of perineural invasion is a strong and independent predictor of local and regional failure in OSCC patients [35–38]. Therefore it is no surprise that patients bearing tumors with this 2/1/EGFR+ expression profile were significantly more likely to recur compared to all of the other IL-1*α*/EGFR expression profiles (Figure 5(b)). Patients with 2/1/EGFR+ tumors also demonstrated worse progression-free survival compared to 2/1/EGFR- tumors (Figures 7(a) and 7(b)). This difference with EGFR expression was not observed in 1/1 (Figures 7(c) and 7(d)) or 1/0 (Figures 7(e) and 7(f)) tumors suggesting a possible interaction between high nuclear IL-1*α* activity and EGFR signaling which is supported by the significant interaction (*p*=0.02) found in Figure 5(b) between the 2/1 IL-1*α* profile score and EGFR+ expression. Of note, we found that 61% of OSCC patients with EGFR+ (score of 2 and 3) tumors experienced tumor recurrence with a median progression-free survival of 13 months (Figure 4(d)). However, if only patients with 2/1/EGFR+ tumors are taken into account, 94% of these patients experienced tumor recurrence with a median survival of 7 months (Figure 7(b)). This suggests that 2/1/EGFR-positivity may be a stronger and more accurate indicator of recurrence and progression-free survival than EGFR expression alone.

At this time, we are unclear as to the role of nuclear IL-1*α* activity in EGFR signaling. Prior reports have shown that nuclear IL-1*α* is involved in transcriptional control by interacting with the histone acetyltransferases p300, PCAF, and Gcn5 [39–41]. Transcription factors important in cancer and proinflammation pathways such as NFkB, Elk-1, C/EBP*β*, or AP-1 are activated by both IL-1 ligands and EGF [42]. EGFR activation has been shown to increase IL-1 ligand expression via increased NFkB activity in breast cancer cells resulting in increased growth and invasion [43]. Also, IL-1 ligands have been reported to transactivate EGFR through a CXCL1-CXCR2 axis [17] and ADAM17 [18] suggesting crosstalk between the EGFR and IL-1 pathways. These reports all support the synergistic interaction between the EGFR and IL-1 pathways but does not fully explain the preferential interaction of EGFR expression with nuclear IL-1*α*. Recently nuclear IL-1*α* has been shown to increase cell proliferation in T-lymphocytic leukemia cells by binding to the promoter region of sp1 leading to increased sp1 expression and activity [15]. Sp1 is a transcription factor involved in cell growth, immune responses, and chromatin remodeling [44]. Sp1 is also involved in the regulation of numerous genes involved in invasion and metastasis [45–47]. EGFR promoter activation requires sp1 and multiple binding sites for sp1 have been discovered [48–50]. This suggests that high nuclear IL-1*α* expression may promote EGFR signaling via sp1 activity which would explain the high recurrence rates observed in patients with 2/1/EGFR+ tumors (Figure 5(b)). Moreover, the IL-1*α* gene has been reported to be regulated by sp1 [51, 52] suggesting a feed-forward relationship between IL-1*α* and sp1 which would ultimately promote EGFR signaling and tumor progression. Further mechanistic studies in this area are necessary to investigate this theory.

## 5. Conclusions

Altogether, we believe we have identified a combined high nuclear IL-1*α*/EGFR+ tumor expression profile as a strong prognostic biomarker for progression-free survival in OSCC patients which warrants further study in other HNSCCs and other EGFR-expressing tumors.

## Figures and Tables

**Table 1:** Patient characteristics by IL-1*α* Immunostaining.

		Nuclear IL-1*α∗*				Cytoplasmic IL-1*α∗*			
Characteristics	Total (n)	2 (%)	1 (%)	0 (%)	*p*-value	2 (%)	1 (%)	0 (%)	*p*-value
Number of Evaluable	141								
Subjects									
Sex									
Male	82	57	58	57	0.99	50	58	59	0.37
Female	59	43	42	43		50	42	41	
Avg Age at Diagnosis									
Male	58	59	58	54	0.74	53	57	62	0.28
(avg (range))	(31-81)	(37-76)	(31-81)	(44-69)			(31-81)	(37-73)	
Female	66	58	68	63		78	67	61	
(avg (range))	(19-33)	(19-81)	(20-93)	(58-73)			(19-93)	(44-80)	
Smoking History									
Active Smoker	56	36	42	29	<0.0001	0	39	44	<0.0001
Never Smoker	49	29	38	14		100	34	33	
Quit <10 Years	13	14	7	29		0	9	11	
Quit >10 Years	17	11	11	29		0	13	11	
Tobacco Chewer	6	11	3	0		0	5	0	
Tumor Site									
Alveolus	21	7	18	0	<0.0001	50	13	19	<0.0001
Floor of the Mouth	31	18	23	29		0	17	44	
Oral Tongue	54	46	37	29		0	44	19	
Other	35	29	23	43		50	26	19	
T Stage									
T1	42	36	29	14	0.001	50	30	26	<0.00001
T2	42	36	28	29		0	30	30	
T3/T4	57	29	42	57		50	39	44	
N Stage									
N0	74	43	56	43	0.137	50	54	48	<0.00001
N1/2a	27	29	16	29		0	16	33	
N2b/2c/3	40	29	28	29		50	30	19	
Differentiation									
well	17	7	13	14	<0.001	0	13	7	<0.0001
moderate	90	75	62	43		50	64	63	
poor	34	18	25	43		50	22	30	
Perineural Invasion									
Yes	69	61	45	57	0.06	0	50	48	<0.001
No	72	39	55	43		100	50	52	
Lymphovascular Invasion									
Yes	52	43	35	43	0.41	50	39	26	0.002
No	89	57	65	57		50	61	74	
Bone Invasion									
Yes	42	21	68	29	<0.001	50	29	30	0.002
No	99	79	32	71		50	71	70	
Radiotherapy									
Yes	78	57	54	71	0.032	100	53	63	<0.00001
No	63	43	46	29		0	47	37	
Chemotherapy									
Yes	22	14	15	29	0.011	50	15	15	<0.00001
No	119	86	85	71		50	85	85	

*∗*Percentages may not sum to 100% due to rounding.

**Table 2:** IL-1*α* expression scores in HNSCC tumors.

IL-1*α* Expression Scores*∗*	Number of Tumors
Nuclear, cytoplasmic	(%)
0/0	3 (2.1)
0/1	4 (2.8)
1/0	23 (16.3)
1/1	81 (57.5)
1/2	2 (1.4)
2/0	1 (0.7)
2/1	27 (19.2)

*∗*0: negative; 1: moderate; 2: strong

**Table 3:** EGFR expression scores in OSCC tumors.

EGFR Expression Score*∗*	Number of Tumors (%)	Number of Recurrences (%)
0	55 (39)	26 (47)
1	15 (11)	6 (40)
2	23 (16)	12 (52)
3	48 (34)	31 (65)

*∗*0: negative; 1: low; 2: moderate; 3: strong

**Table 4:** Postsurgery therapy in OSCC patients.

EGFR*∗*/IL-1*α* Expression Profile	Number of Patients	Chemotherapy (%)	Radiation (%)	Chemotherapy+Radiation (%)	P-value
2/1/EGFR+	*16 *	*3 (19)*	*10 (63)*	*3 (19)*	*0.19*
2/1/EGFR-	*11*	*1 (9)*	*6 (55)*	*1 (9)*	
1/1/EGFR+	*39*	*7 (18)*	*17 (44)*	*7 (18)*	*0.10*
1/1/EGFR-	*42*	*5 (12)*	*24 (57)*	*5 (12)*	
1/0/EGFR+	*8*	*0 (0)*	*4 (50)*	*0 (0)*	*0.008*
1/0/EGFR-	*15*	*2 (13)*	*10 (67)*	*2 (13)*	

*∗*EGFR+: strong (3)+moderate (2) expression

EGFR-: low (1)+negative (0) expression

## Data Availability

The datasets generated during and/or analyzed during the current study are not publically available due to privacy and other restrictions but are available from the relevant authors on reasonable request.
